# Dystonic tremor in Writer’s cramp Mimicking primary handwriting tremor

**DOI:** 10.1016/j.prdoa.2025.100410

**Published:** 2025-11-26

**Authors:** Paulo Cataniag, Cezar Thomas Suratos, Cid Czarina Diesta, Jed Noel Ong

**Affiliations:** a MakatiMed Institute of Neurological, Neurosurgical and Behavioral Sciences (M.I.N.D.S.), Section of Movement Disorders, Makati Medical Center, Makati City, Philippines; b Department of Neurosciences, Baguio General Hospital and Medical Center, Baguio City, Philippines

**Keywords:** Writer’s cramp, Task-specific dystonia, Dystonic tremor, Primary writing tremor

## Abstract

**Introduction:**

Writer’s cramp is a task-specific focal dystonia with peculiarity of occurring only during handwriting. The presence of abnormal posturing and dystonic tremor may exhibit distinctive patterns across individuals, and may closely resemble primary writing tremor, especially when dystonic postures are mild.

**Case description:**

We present a 48-year-old right-handed Filipino businessman who developed a gradually worsening right-hand tremor confined to handwriting over one year, stabilizing after six months. Neurological examination was otherwise normal, but writing tests revealed dystonic wrist extension and shoulder abduction, along with wrist rotational/pronation-supination dystonic tremor during writing. Wavy/oscillating drawing patterns were observed. Diagnosis of writer’s cramp was made, which mimicked primary writing tremor due to overlapping features. Multiple oral medications were ineffective, but ultrasound-guided botulinum toxin injections led to clinical improvements, with better handwriting during faster writing and worse at slower speeds.

**Conclusion:**

This case underscores the complexity of diagnosing and treating task-specific dystonia with tremor. Incorporating tests like line-drawing at different speeds, including vertical and diagonal lines, can enhance evaluation.

## 1 Introduction

Dystonia is a movement disorder involving sustained or intermittent abnormal, patterned, and often repetitive movements or postures [1]. When such dystonic movements specifically occur during the execution of a specific skilled motor task, it is termed task-specific focal dystonia. One example of this intriguing group of disorders is writer’s cramp (WC) that occurs during handwriting [2]. In more complex cases, the dystonic symptoms can involve the dominant hand during additional fine motor tasks [3]. WC has a prevalence of 14 (11–17) per million, typically manifesting in the 4th to 5th decades of life [4,5]. Men are more frequently affected than women, although women tend to have an earlier onset of symptoms [6].

Abnormal posturing of the fingers, hand, or forearm is observed and may exhibit distinctive patterns across individuals [6]. WC can initially present as an extremely task-specific problem, affecting only a single letter or number, which may be mistaken for a psychogenic disorder [7]. Typically, other aspects of hand function remain intact, and neurological examination findings are generally normal [7]. Patients complain about decreased dexterity, discomfort, and fatigue in the affected hand, potentially leading to a reduced quality of life [2,8,9]. History of prolonged and repetitive hand use is often associated with the development of WC [2]. The pathogenic mechanisms underlying this disease include abnormal cortical excitability and dysfunctional motor preparation during the preparatory phase of writing [10].

When dystonic tremor presents alongside WC, it can resemble primary writing tremor (PWT), particularly when dystonic features are mild. PWT is an action tremor that occurs only during writing activities [11], similar in task specificity to WC. The overlap between these two conditions has not been extensively documented in the literature. Therefore, we present and describe a case of a Filipino adult male with WC presenting with a dystonic tremor that mimics PWT, along with the lessons learned from this case.

## 2 Case description

A 48-year-old right-handed Filipino businessman presented with a one-year history of gradually worsening involuntary tremor of the right hand during writing. He was able to hold the pen normally in a writing posture. However, the tremor appeared as soon as he started to write, with no specific letters or numbers triggering it. The pen jerked rapidly across the paper, impairing handwriting legibility. Initially mild, the tremor became more noticeable over time. The patient did not exert extra force or grip tightly on the pen. No involuntary movements occurred at rest or during other hand activities. Writing with the left (non-dominant) hand did not reproduce the tremor.

A neurologist diagnosed handwriting tremor after one month. Propanolol and Clonazepam were tried but ineffective, and varying pen thicknesses did not help. The tremor gradually worsened but remained confined to writing, affecting handwriting quality and causing mild tightness on the right hand and forearm. The patient temporarily ceased treatment and avoided writing, reaching a symptom plateau after six months.

He then sought further evaluation at the Movement Disorders Clinic due to the impact on his political aspirations, stemming from poor handwriting. There were no relevant past medical, trauma, medication, or toxin exposures, and no family history of movement disorders. He had no history of chronic and repetitive hand use, and his review of systems was otherwise unremarkable.

**Fig. 1:**
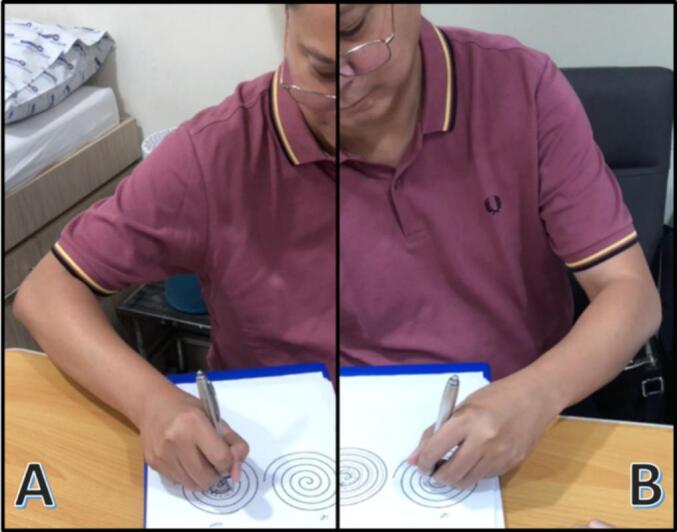
**A–B. Hand and arm posturing during handwriting**. Image A shows right dystonic wrist extension and shoulder abduction, as compared with the normal writing postures in Image B.

The physical and neurological examination were unremarkable, but the writing tests revealed several abnormalities. Hand posture was normal at rest, with the patient holding the pen in a typical writing manner without abnormal posturing of the fingers or wrist. The thought of writing did not provoke abnormal movements. During the writing assessment, the patient showed tremulous rotational/pronation-supination movements of the right wrist, with wrist extension and shoulder abduction. However, finger and elbow positions remained normal. No sensory tricks or alleviating maneuvers were noted. No abnormal posturing was observed in the left hand at rest or during tasks. No mirror movements were detected when writing with the opposite hand. *See* Fig. 1 *and* video*s in link*: https://drive.google.com/drive/folders/1Tf8XeADDHRht7zTQkxayhG0i-O1U0M26?usp=sharing.

**Fig. 2:**
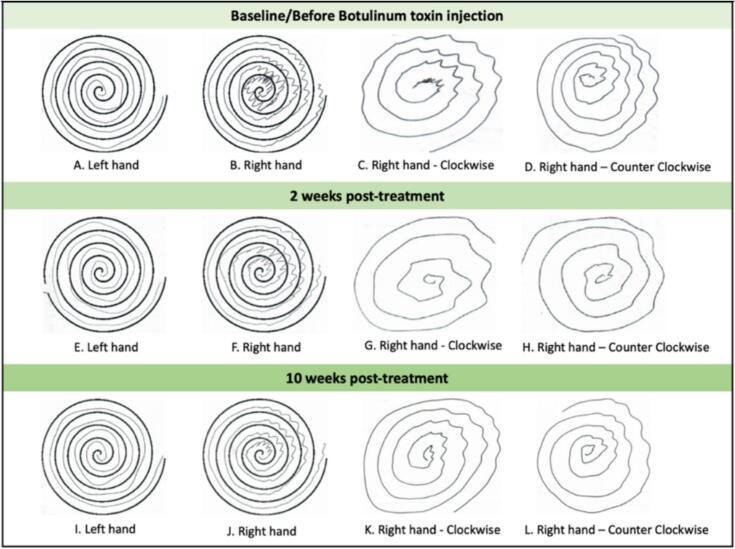
**A–L: Spiral drawings of both hands before and after BoNT injection.** Images A and B depict spiral drawings of both hands within the archimedean spiral, with the wrist not touching the paper. Images C and D images show spiral drawings of the affected (right) hand in clockwise and counter clockwise directions. Images E–H are the corresponding drawings obtained 2 weeks post-treatment, while Images I–L are those obtained after 10 weeks.

**Fig. 3:**
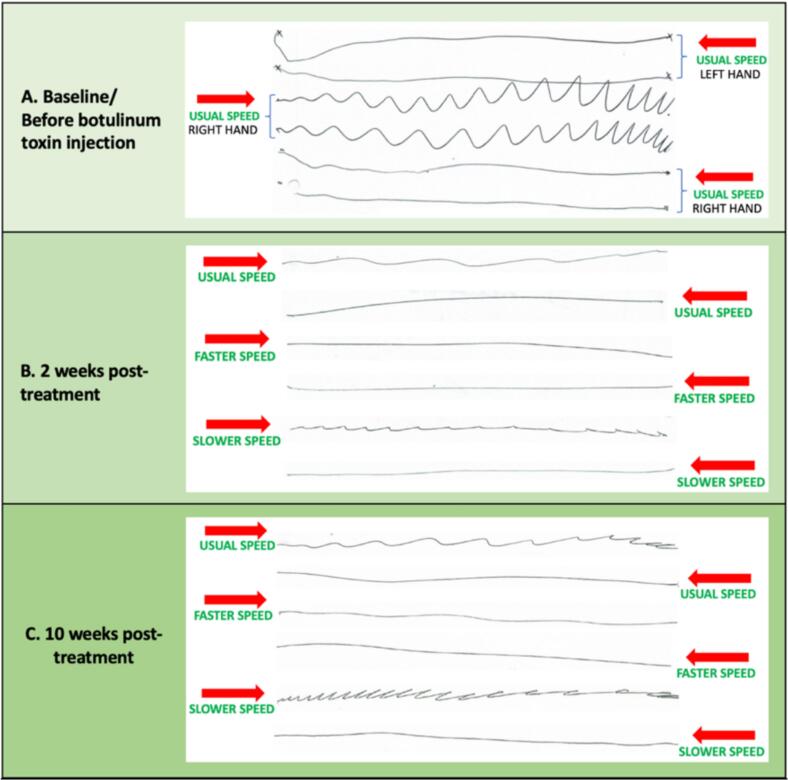
**A–C**. **Horizontal line drawing test before and after BoNT injection**. Red arrows point the direction of drawing. Image A shows a wavy pattern confined to the right hand during rightward drawing at the usual speed. Images B and C display improvement in the right hand pattern after 2 and 10 weeks of treatment, with reduced amplitude during usual and slow speeds, and a complete resolution during faster writing. (For interpretation of the references to colour in this figure legend, the reader is referred to the web version of this article.)

**Fig. 4:**
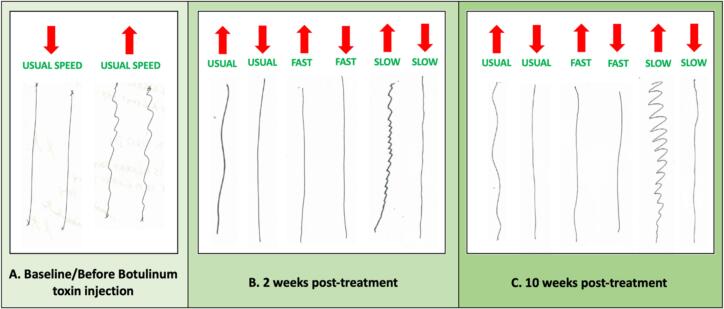
**A–C**. **Vertical line drawing tests of the right hand before and after BoNT injection**. Red arrows point the direction of drawing. Image A shows a wavy pattern during upward drawing at the usual speed. Image B demonstrates of the upward wavy pattern during both usual and faster speeds after 2 weeks of treatment. Image C depicts worsening of the upward pattern at 10 weeks post-treatment, as the treatment effects diminished. (For interpretation of the references to colour in this figure legend, the reader is referred to the web version of this article.)

**Fig. 5:**
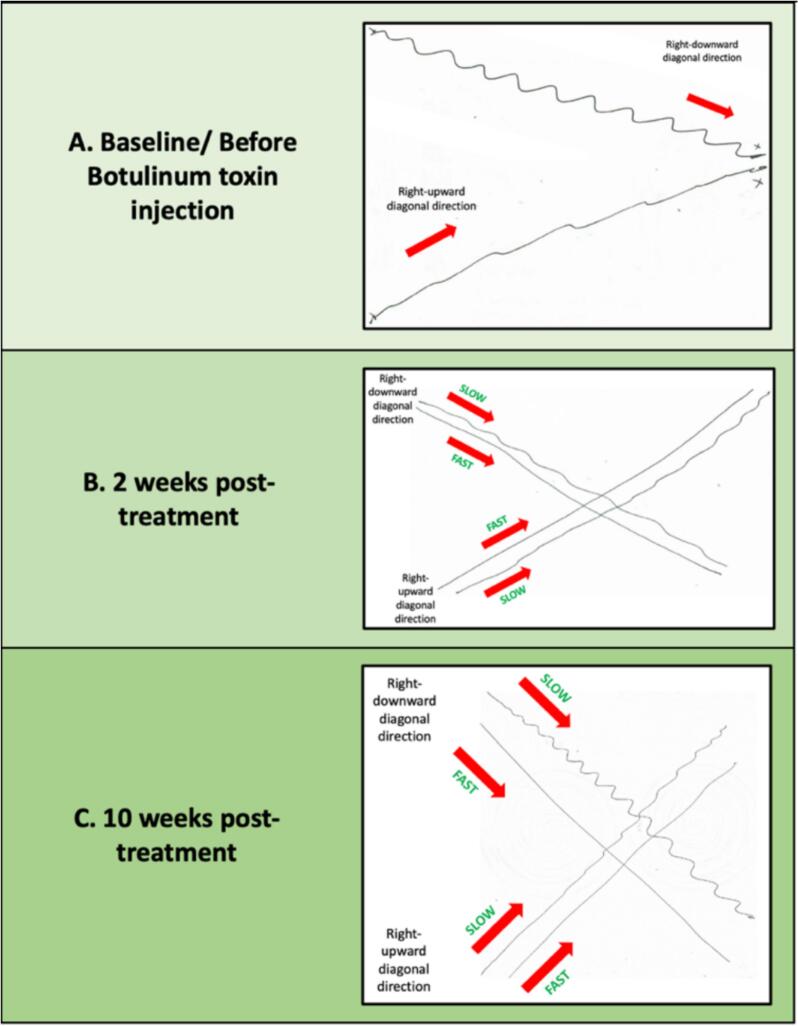
**A–C. Diagonal line drawing tests of the right hand before and after BoNT injection**. Red arrows point the direction of drawing. Image A shows a wavy pattern during both upward and downward directions at the usual speed, with the pattern being during downward drawing. Image B demonstrates improvement in both patterns during faster and slower speeds after 2 weeks of treatment. Image C depicts worsening of the downward pattern during slow drawing at 10 weeks post-treatment, as the treatment effects diminished. No patterns were drawn at usual speeds in Images B and C. (For interpretation of the references to colour in this figure legend, the reader is referred to the web version of this article.)

**Fig. 6:**
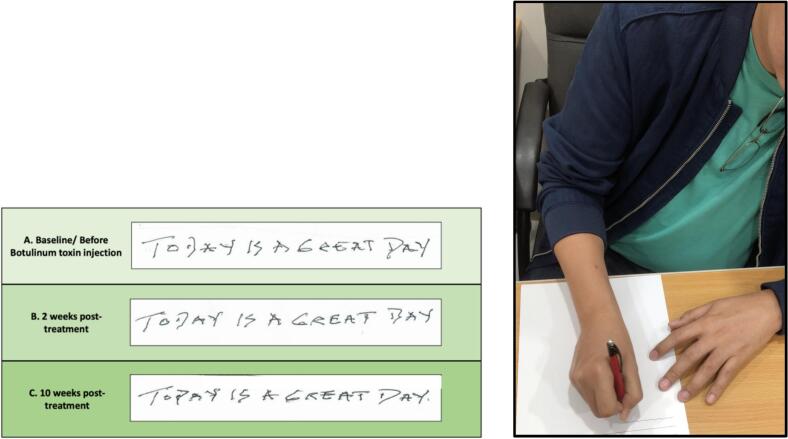
**A–C. Penmanship.** Images show the penmanship of patient from baseline up to 2 and 10 weeks after BoNT treatment.

The patient exhibited variable pressure on the paper, sometimes slightly increased or faint. Wavy or oscillating patterns in the Archimedes spiral were noted between the 12 and 3o'clock positions, in both clockwise and counter clockwise directions (*see* Fig. 2A–D). Similar patterns were observed during rightward horizontal lines (*see* Fig. 3A), upward vertical lines (*see* Fig. 4A), and rightward diagonal lines (*see* Fig. 5A) at patient’s usual drawing speed. These patterns were absent during the opposite linear directions. Penmanship was partially legible, with some waviness in letters (*see* Fig. 6A). He did not display a tight grip on the pen.

Blood tests and cranial MRI were normal, and nerve conduction studies showed normal nerve function. However, monopolar needle electromyography (EMG) performed during writing revealed abnormal findings consistent with focal dystonia in writer’s cramp, evidenced by irregular bursts of high-amplitude motor unit potentials (MUAPs), particularly in muscles such as the flexor carpi radialis, extensor carpi radialis, brachioradialis, and less frequently in the flexor digitorum profundus. Surface EMG, genetic testing and kinematic analysis were not available.

The patient was diagnosed with writer’s cramp primarily due to the dystonic wrist extension, which likely caused the dystonic tremor, and accompanied by shoulder abduction as an overflow dystonia. As the patient previously showed no response to propranolol and clonazepam, levodopa-carbidopa, biperiden and baclofen were also attempted but did not alleviate the symptoms. Other medications like trihexyphenidyl, primidone, and tetrabenazine were not available locally. As a result, onabotulinum toxin A injections were given under ultrasound guidance, targeting the right wrist extensors—the extensor carpi ulnaris and extensor carpi radialis—with 10 units in each muscle.

**Fig. 7:**
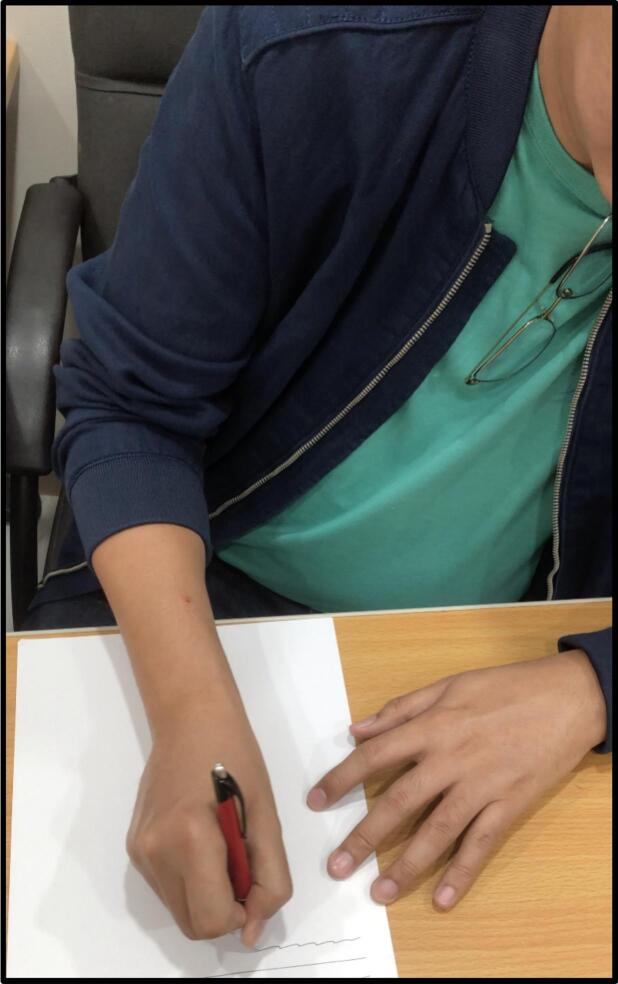
Images shows improvement of dystonic right wrist extension and shoulder abduction after BoNT treatment.

Two weeks following chemodenervation with botulinum neurotoxin (BoNT), the patient showed improvement in dystonic wrist extension and shoulder abduction while writing (see Fig. 7 and see video link). Improvements were also observed in the spiral drawings in both clockwise and counterclockwise directions (*see* Fig. 2G and H). To evaluate the effect of speed, straight line drawing tests were performed at the usual, slower, and faster drawing speeds. Subsequent observations indicated further improvement during faster drawing speeds and worsened patterns during slower speeds (*See* video *link*). These speed-dependent changes were evident across horizontal, vertical, and diagonal lines (*see* Fig. 3, Fig. 4, Fig. 5). Penmanship remained unchanged (*see* Fig. 6B). Subjective improvement was 30 %. The only adverse effect was mild wrist extension weakness, which resolved spontaneously within a few weeks. At ten weeks post-treatment, signs of a return to baseline patterns emerged due to diminishing effects of BoNT (s*ee* Fig. 2, Fig. 3, Fig. 4, Fig. 5). Penmanship continued to be unaffected (*see* Fig. 6C).

## 3 Discussion

Skilled hand movements represent the peak of human motor development, and dedicated practice can result in extraordinary dexterity with societal significance [12]. Writing involves complex, well-coordinated movements of multiple muscles in the hand and forearm, and the fluidity of writing depends on precise muscle synchronization, allowing for rapid, smooth strokes [13]. While prolonged and repetitive hand use is often associated with WC, any change in this normally stable and learned pattern can also serve as a major trigger for dystonic symptoms [12]. The more a task deviates from the limb's natural ability, the higher the risk of developing dystonia [12]. However, it is notable that our patient does not have a history of prolonged or repetitive hand activity, recent changes in hand use or any hand trauma.

Dystonic movements can spread or “overflow” to nearby unaffected body parts, producing excessive, unintended actions unrelated to the voluntary movement [1]. The patient’s dystonic posturing does not appear to be compensatory movements aimed at reducing the tremor. Instead, we believe that wrist extension was the primary dystonic movement, while shoulder abduction was an overflow dystonia. The fact that shoulder abduction improved—despite no injections being administered to the deltoid muscles—suggested this was an overflow dystonia originating from the primary wrist extension dystonia.

The tremulous handwriting was likely caused by a dystonic tremor (DT) during writing. DT is a postural or kinetic tremor characterized by irregular amplitudes and varying frequencies (primarily below 7 Hz) that occur in the body region affected by dystonia [14,15]. This differs from “tremor associated with dystonia”, a type of tremor that affects a body part unaffected by dystonia [11]. DT typically worsens when the patient actively moves the affected limb against the direction of dystonic pull, but can diminish or disappear when the limb is positioned in alignment with the dystonia [14].

However, in our patient, the opposite was observed: tremors appeared during writing motions involving more wrist extension (same direction as the dystonic pull), and not during wrist flexion. These included spiral drawing at 12–3o’clock and straight lines in specific directions (rightward horizontal, upward vertical, and right-downward diagonal). Movements in other directions, which involved more wrist flexion, elicited little to no tremor, as reflected in the drawing patterns. This could represent a null point of the dystonia, which is a body position that elicits no dystonic tremor. Additionally, the dystonic wrist extension produced a rotational/pronation-supination tremor rather than the expected flexion–extension tremor. This suggests that the underlying mechanism of our patient's DT is more complex. The wrist extensor muscles were initially targeted for BoNT injections in an effort to control the tremor.

Mild, task-specific dystonic contractions may be overlooked, leading to misdiagnosis as PWT, while dystonic tremors in WC can closely resemble PWT. In one study, tremulousness of the hand while writing was present in 28.8 % of participants [6]. The classification of PWT has been widely debated for decades, as some consider it a form of dystonia, while others argue it is a variant of essential tremor (ET). However, in 2018, a consensus statement declared that task- and position-specific tremors are no longer classified as variants of ET [11]. Additionally, it is now widely accepted that tremor is part of the phenotypic spectrum of dystonia [16]. Our patient’s DT exhibits features that aligns more closely with WC than PWT, such as unilateral or asymmetric presentation, focal task- or position-specific nature, irregular amplitude and frequency, abnormal posturing, overflow to nearby body parts, presence of null point and non-responsiveness to Propanolol. Other features of DT, such as mirror dystonia and response to sensory tricks or alleviating maneuvers [1,14], were not present in our patient. However, in one study, only 46 % of patients with WC presented with mirror dystonia [3]. Furthermore, needle EMG demonstrated irregular, high-amplitude MUAP bursts, a pattern more consistent with dystonia, whereas primary tremor typically displays regular, rhythmic discharges during writing.

Handwritten spirals often show a single dominant tremor axis in ET (e.g. right hand tremor being most severe at 1–2o’clock and 7–8o’clock), while multidirectional tremors are more typical of dystonic tremor. However, this method has only moderate diagnostic reliability, with a sensitivity of 68 % and specificity of 60 % [17]. In our patient, the tremor was at 12–3o’clock, with no tremor seen in the opposite axis (6–9o’clock).

WC causes reduced writing speed, disrupted flow, and variable pen pressure [18], all of which were observed in our patient. Interestingly, following BoNT injection, his handwriting worsened at slow speeds but improved at faster speeds—an opposite pattern compared to previous studies [19]. This variability underscores the clinical complexity and heterogeneity among WC patients. One possible explanation is that rapid movements allow motor control to bypass abnormal basal ganglia circuits and instead rely more on cerebellar feed-forward mechanisms [20]. Furthermore, the botulinum toxin alters muscle spindle input and gamma-motor control, so movements that require slow, precise sensory feedback may trigger excessive corrective responses [21].

Genome-wide studies have linked the arylsulfatase G (ARSG) gene to writer’s cramp, although no definitive causative mutation has yet been identified [22]. Genetic testing is not available locally in the Philippines; however, a positive result is unlikely to change management, and the patient showed no features suggestive of an underlying genetic disorder. Additionally, kinematic analysis, which could provide insights into handwriting mechanics such as fluency and speed, is not accessible.

The dose and specific muscle targets will be adjusted during future botulinum toxin injection sessions, guided by the patient’s response and satisfaction. If botulinum toxin therapy becomes suboptimal, the patient will be advised to increase the use of keyboards to bypass WC or to switch writing to the other hand, although in some cases, the task-specific dystonia eventually affects the opposite hand [8,23].

Current management options for WC include oral medications, BoNT injections, surgical procedures, and physical therapy [24]. Since our patient did not respond to oral medications, BoNT injections were administered, as they are well-established as safe and effective for more than a decade [5]. Physical/occupational therapy can amplify the beneficial effects of BoNT therapy [25]. A recent study found that combined sensory-motor rehabilitation has sustained effectiveness [26]. Although focal BoNT injections can improve symptoms, they have temporary effects; thus, when disability from WC surpasses the risks associated with invasiveness, surgical interventions can be considered, such as deep brain stimulation or stereotactic ablation surgery [27,28]. Additionally, a simple and cost-effective alternative is the use of writing orthotic device [29]. Recently, Zolpidem has shown promise in improving symptoms [30].

## 4 Recommendations

Care and diligence must be observed in the assessment of WC. Scales specifically designed to objectively evaluate WC, such as the Writer’s Cramp Rating Scale (WCRS), Writer’s Cramp Impairment Scale (WCIS), and Writer’s Cramp Disability Scale (WCDS), may be useful in future cases. Incorporating a vertical and diagonal line test into formal assessments is also recommended. Adding line drawing tests at both slow and fast speeds could also provide additional information, though standardization of speed remains challenging.

## Ethical approval

This case report was conducted in accordance with ethical standards. Patient consent was obtained for publication of this case report.

## CRediT authorship contribution statement

**Paulo Cataniag:** Writing – original draft, Investigation, Conceptualization. **Cezar Thomas Suratos:** Writing – review & editing, Supervision, Investigation, Conceptualization. **Cid Czarina Diesta:** Writing – review & editing, Supervision. **Jed Noel Ong:** Writing – review & editing, Supervision, Investigation, Conceptualization.

## Funding

None.

## Declaration of competing interest

The authors declare that they have no known competing financial interests or personal relationships that could have appeared to influence the work reported in this paper.
